# Dexmedetomidine exerts an anti-inflammatory effect *via* α2 adrenoceptors to alleviate cognitive dysfunction in 5xFAD mice

**DOI:** 10.3389/fnagi.2022.978768

**Published:** 2022-09-20

**Authors:** Su-mei Luo, Long-yan Li, Li-zhe Guo, Lu Wang, Yan-feng Wang, Na Chen, E. Wang

**Affiliations:** ^1^Department of Anesthesiology, Xiangya Hospital Central South University, Changsha, China; ^2^National Clinical Research Center for Geriatric Disorders (Xiangya Hospital), Changsha, China

**Keywords:** Alzheimer’s disease, dexmedetomidine, yohimbine, amyloid plaques, neuroinflammation

## Abstract

**Background:** Inflammation promotes the progression of Alzheimer’s disease (AD). In this study, we explored the effect of dexmedetomidine on inflammation and cognitive function in a mouse model of AD.

**Methods:** 5xFAD mice were intragastrically administered saline, dexmedetomidine, or dexmedetomidine and yohimbine for 14 days. The effects of dexmedetomidine on the acquisition and retention of memory in the Morris water-maze test and Y maze were evaluated. The deposition of amyloid beta protein (Abeta) and cytokine levels in the hippocampus were assessed. The expression of Bace1 protein and NFκB-p65 protein was assessed by Western blotting.

**Results:** Compared with WT mice, 5xFAD mice exhibited cognitive impairment in the Morris water maze test and Y maze test. Cognitive decline was alleviated by dexmedetomidine and this was reversed by the α2 adrenoceptor antagonist yohimbine. Compared with saline treatment, dexmedetomidine led to a reduction in the Abeta deposition area (*p* < 0.05) and in the mean gray value (*p* < 0.01) in the hippocampus of 5xFAD mice. Compared with saline treatment, dexmedetomidine inhibited the activation of astrocytes and microglia in the hippocampal DG of 5xFAD mice and reduced the area of GFAP (*p* < 0.01) and IBA1 (*p* < 0.01). The level of IL-1β in the hippocampus decreased significantly after dexmedetomidine treatment compared with saline treatment in 5xFAD mice (*p* < 0.01). Yohimbine neutralized the effects of dexmedetomidine. Dexmedetomidine inhibited the expression of BACE1 and NF-κB p65 (*p* < 0.01), and these changes were reversed by yohimbine treatment.

**Conclusion:** Dexmedetomidine alleviates cognitive decline, inhibits neuroinflammation, and prevents the deposition of Abeta in 5xFAD mice. The effect is mediated by the α2 adrenoceptor-mediated anti-inflammatory pathway. Dexmedetomidine may be effective for the treatment of AD and a better choice for the sedation of AD.

## Introduction

Alzheimer’s disease (AD) results in memory impairment and cognitive decline (Thies and Bleiler, 2012). AD is characterized by the pathogenesis of extracellular amyloid plaques, neurofibrillary tangles, hyperphosphorylated tau protein, reductions in synaptic density, and neuroimmune activation (Bertram and Tanzi, 2008). Neuroinflammation is an important factor in AD (Parhizkar and Holtzman, 2022), persistent neuroinflammation induces amyloid-beta (Aβ), and neurofibrillary tangle production and death of neurons (Rajesh and Kanneganti, 2022; Weng et al., 2022), activated microglia and astrocytes cause the release of inflammatory factors, such as IL-6, IL-1β, and NF-κB (Bales et al., 1998). Many studies revealed that inflammatory factors involve the Aβ deposition and tau protein phosphorylation and direct toxic effects on neurons and synapses (Heneka et al., 2015; Hong et al., 2016; Sung et al., 2020), leading to the decline of cognitive function. Thus far, therapeutic options for AD are very limited.

Dexmedetomidine is an α2-adrenergic receptor agonist that is commonly used clinically for sedation and analgesia. In recent years, it has been reported that dexmedetomidine can protect neurocognition by reducing interleukin (IL)-6 and IL-1β in the hippocampus (Wang et al., 2019; Mei et al., 2021), it also can reduce the inflammatory response by reducing IL-1β and NF-κB levels *via* α2 adrenoceptors (Li et al., 2020). In addition, Chen’s report indicates that NF-κB overexpression leads to upregulated β-secretase cleavage and Abeta production (Chen et al., 2012). We hypothesized that dexmedetomidine can delay cognitive decline in 5xFAD mice and explored its mechanism.

## Materials and Methods

### Animals and ethics statement

All animal experimental procedures were approved by the Ethics Committee for Animal Research of Central South University and were performed in accordance with guidelines for treating laboratory animals. Furthermore, the study conformed with the National Institutes of Health Guide for the Care and Use of Laboratory Animals (NIH Publication No. 8023, revised 1978). For this study, we used the transgenic mouse line 5xFAD, which expresses five human mutations in APP and PS1 [B6SJL-Tg (APP*K670N*M671L*I716V*V717I, PSEN1*M146*L286V) 6799Vas/J], through a neuron-specific element in the Thy1 promoter. 5xFAD mice were originally gifted from the Shen Laboratory (School of Life Science, University of Science and Technology of China). The animals were housed with free access to food and water in a colony room under the following conditions: a temperature of 19°C–22°C humidity level of 40%–60%, and a 12 h/12 h cycle of automatic lighting. For genotyping of 5xFAD mice by PCR, the following primer pairs were used: forward primer (5’-AGG ACT GAC CAC TCG ACC AG-3’) and reverse primer (5’-CGG GGG TCT AGT TCT GCA T-3’).

### Treatment of 5xFAD mice with dexmedetomidine

Forty-eight male 22-week-old wild-type and 5xFAD mice were divided into four groups: the wild-type treated with normal saline group (WT+NS), 5xFAD treated with normal saline group (AD+NS), 5xFAD treated with dexmedetomidine group (AD+Dex), and 5xFAD treated with dexmedetomidine and yohimbine group (AD+Dex+Y). The mice in the dexmedetomidine group were given dexmedetomidine (Hengrui Pharmaceutical Co. Ltd, Jiangsu, China) intragastrically at a dosage of 100 μg/kg once a day for 14 days. The mice in the dexmedetomidine and yohimbine groups were given the same dosage of dexmedetomidine and a dose of 0.5 mg/kg yohimbine hydrochloride (Abmole, Houston, TX), yohimbine was given 10 min before dexmedetomidine. An equal volume of saline was given intragastrically to mice in the control groups.

### Morris water maze behavioral tests

After 14 days of continuous feeding, the mice were brought to the testing room to adapt to the environment for 30 min before the behavioral test. The Morris water maze (MWM) test was performed with minor adjustments as previously described. The test consisted of 5 days of learning trials and a 1-day probe test. The mice were given 1 min to find the platform. If a mouse found the platform within 1 min and stayed for 3 s, we considered the mouse to have successfully found the platform and ended the experiment. Otherwise, something was used to guide the mouse to the platform and stay for 20 s. In the probe test, the platform was removed, and the mice were allowed to swim for 1 min. All mouse movements were recorded and analyzed by Smart v3.0 (Panlab, Spain), a computerized tracking system.

### Y-maze behavioral tests

Mice were brought to the testing room to adapt to the environment for 30 min before the test and then subjected to the Y-maze test, as previously described (Dinel et al., 2014; Jeon et al., 2018). The Y-maze consists of three arms and a center, which together form a Y shape. Before the test, one arm of the maze was closed, and then the mice were placed in one open arm facing the wall. Mice were allowed 8 min to explore the two open arms of the maze. Thirty minutes later, the mice were returned to the Y-maze with all arms open for 5 min. The time spent in the novel arm and the new arm entries were recorded.

### Real time RT-PCR analysis

After behavioral testing, the mice were euthanized by cervical dislocation, and the hippocampus was immediately harvested. RNA was extracted from the hippocampus using TranZol Up (TRANS, China) according to the manufacturer’s instructions. A NanoDrop (Thermo, Wilmington, DE) was used to quantify RNA, and the SureScript First-Strand cDNA synthesis kit (Genecopoeia, Rockville, MD) was used to reverse transcribe cDNA. Real-time RT-PCR was performed using a 96-well microfluidic card, and gene expression was assessed by performing SYBR-green reagent assays (Applied Biosystems, Thermo Fisher Scientific, Wilmington, DE). The β-actin gene was used to normalize the expression of target genes. The primer sets for RT-qPCR analysis were as follows: β-actin: forward primer (sequence 5’−3’) (TGCTCTCCCTCACGCCATCC); reverse primer (sequence 5’−3’) (GTCACGCACGATTTCCCTCTCAG); Bace1: forward primer (sequence 5’−3’) (CAGTGGAAGGTCCGTTTGTT); reverse primer (sequence 5’−3’) (CTAAAGGATGCTGGGCAGAG).

### Immunofluorescence staining assay

Immunofluorescence staining was performed on frozen coronal sections of the mouse brain. The brain sections were washed with PBS and incubated with Immunol Staining Blocking Buffer (Beyotime Biotechnology, China) to block nonspecific reactions for 2 h at room temperature. After blocking, the sections were incubated overnight at 4°C in Immunol Staining Primary Antibody Dilution Buffer (Beyotime Biotechnology, China), which contained rabbit anti-β-amyloid antibody (1:1,000, Cell Signaling Technology, Boston, MA). The next day, sections were incubated for 2 h at room temperature in PBS containing Alexa Fluor 488 (1:500, green, Invitrogen, CA). Finally, the sections were photographed using a Nikon fluorescence microscope. The results were analyzed with ImageJ software.

### Western blot

Tissues were homogenized in RIPA buffer supplemented with protease and phosphatase inhibitors (NCM Biotech, China), sonicated to shear DNA, and centrifuged at 14,000× *g* for 20 min at 4°C. Equal amounts of protein sample (50 μg) were resolved by SDS-PAGE. The following primary antibodies were used: rabbit anti-β-actin antibody (1:10,000; Affinity, Cincinnati, OH), rabbit anti-bace1 antibody (1:1,000; Cell Signaling Technology, Boston, MA), rabbit anti-NF-KB antibody (1:1,000; Cell Signaling Technology, Boston, MA), and goat anti-rabbit IgG (H+L) HRP (1:5,000; Affinity, Cincinnati, OH). Quantification of immunoreactive bands is reported as a ratio against β-actin using ImageJ software.

### Enzyme-linked immunosorbent assay

Hippocampal samples were collected from each group for use in assessing the effects of dexmedetomidine on the hippocampal levels of inflammatory factors. An IL-6 ELISA kit (Cusabio Biotech Co., Ltd, Hubei, China) and IL-1β ELISA kit (Proteintech Group Inc., Rosemont, PA) were used, and the ELISA protocol for IL-6 and IL-1β was performed according to the respective instructions.

### Statistical analysis

All data are expressed as the means ± SD or means ± SEM and were analyzed with GraphPad Prism 8.0. One-way ANOVA or two-way ANOVA was used for comparisons with three groups or more. *P* < 0.05 was considered indicative of statistical significance.

## Results

### Dexmedetomidine protects against cognitive impairment in 5xFAD mice

**Figure 1:**
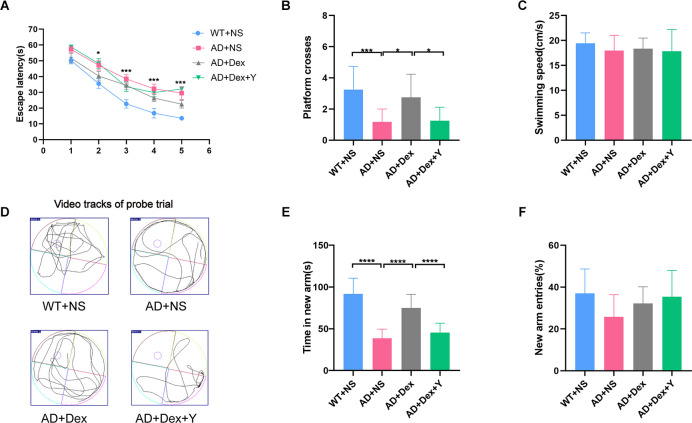
Dexmedetomidine improves learning and memory ability in 5xFAD mice. The data from the Morris water maze **(A–D)** and Y maze **(E,F)** were recorded and statistically analyzed. Panel **(A)** represents latency during the acquisition phase (means ± SEM, two-way repeated measures ANOVA and Bonferroni *post-hoc* test, *p < 0.05, AD+NC compared to the WT+NC group). Panel **(B)** represents the number of animals crossing the platform. Panel **(C)** represents swimming speed by mice in the target quadrant during the probe trial. Panel **(D)** represents video tracks of the probe trial. Panel **(E)** represents time spent in the new arm of the Y maze. Panel **(F)** represents the percentage of new arm entries in the Y maze (means ± SEM, ANOVA, Tukey’s test, *p < 0.05, ***p < 0.001, ****p < 0.0001). *n* = 12 in each group.

In the Morris water maze, mice in the 5xFAD treated with normal saline (AD+NS) group spent more time finding the escape platform than mice in the WT+NS group (*p* < 0.05). There is no statistically significant difference between the AD+NS group and the AD+Dex group, nor between the AD+Dex group and the AD+Dex+Y group. But the trends can be seen as follows, mice in the AD+NS group spent more time finding the platform than mice in the 5xFAD treated with dexmedetomidine (AD+Dex) group, and mice in the 5xFAD treated with dexmedetomidine and yohimbine (AD+Dex+Y) group spent more time finding the platform than mice in the AD+Dex group (Figure 1A). In the probe test, mice in the wild-type treated with normal saline (WT+NS) group crossed the platform more often than mice in the AD+NS group (WT+NS vs. AD+NS, 3.25 ± 0.43 vs. 1.17 ± 0.24, *p* < 0.001). Mice in the AD+Dex group crossed the platform more often than those in the AD+NS group and AD+Dex+Y group (AD+Dex vs. AD+NS, 2.75 ± 0.43 vs. 1.17 ± 0.24, *p* = 0.01), (AD+Dex vs. AD+Dex+Y, 2.75 ± 0.43 vs. 1.25 ± 0.25, *p* = 0.02, *n* = 12 in each group; Figure 1B). There was no significant difference in swimming speed among the four groups (Figure 1C). Video tracks of the probe trial can be seen in Figure 1D.

In the Y maze, mice in the WT+NS group spent more time in the new arm than mice in the AD+NS group (WT+NS vs. AD+NS, 91.67 ± 5.51 vs. 38.79 ± 3.11 s, *p* < 0.001). Mice in the AD+Dex group spent more time in the new arm than mice in the AD+NS group (AD+Dex vs. AD+NS, 74.95 ± 4.72 vs. 38.79 ± 3.11 s, *p* < 0.001). Furthermore, mice in the AD+Dex group spent more time in the new arm than mice in the AD+Dex+Y group (AD+Dex vs. AD+Dex+Y, 74.95 ± 4.72 vs. 45.46 ± 3.25 s, *p* < 0.001, *n* = 12 in each group; Figure 1E). There was no significant difference in new arm entries among the four groups (Figure 1F).

### Dexmedetomidine alleviates the deposition of Abeta in the hippocampus of 5xFAD Mice

**Figure 2:**
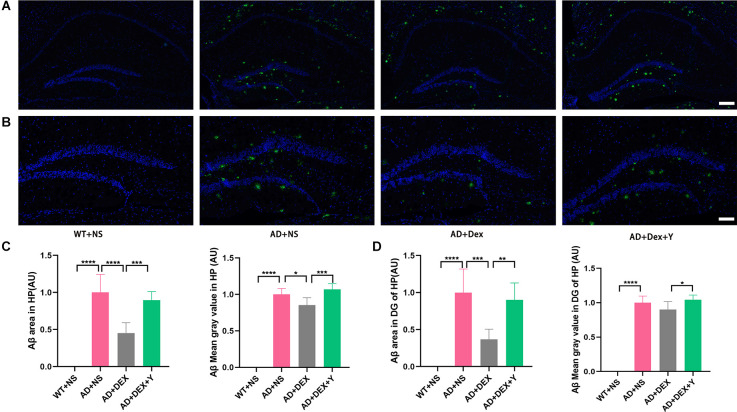
Dexmedetomidine alleviates the deposition of Aβ **(A,B,C,D)** (means ± SD, ANOVA, *p < 0.05, **p < 0.01, ***p < 0.001, ****p < 0.0001). **(A)** Immunofluorescence staining of hippocampal sections from WT and 5xFAD mice, scale bars, 200 μm. **(B)** Immunofluorescence staining of the hippocampal DG sections from WT and 5xFAD mice, scale bars, 100 μm. **(C)** Bar graph represents Abeta expression levels in the hippocampus. **(D)** The bar graph represents Abeta expression levels in the hippocampus DG.

Abeta deposition was detected in the four groups of mice with an immunofluorescence staining assay (Figure 2A). There was no Abeta deposition in the hippocampus of the WT+NS group. Mice in the AD+NS group and AD+Dex+Y group had larger areas of Abeta deposition in the hippocampus, especially in the hippocampal dentate gyrus (DG; Figure 2B) than mice in the AD+Dex group. The Abeta deposition areas in the hippocampus in different groups were as follows: AD+NS vs. AD+Dex, 1.00 ± 0.24 vs. 0.45 ± 0.14 AU, *p* < 0.001; AD+Dex vs. AD+Dex+Y, 0.45 ± 0.14 vs. 0.89 ± 0.11, *p* < 0.001. The mean gray values of Abeta deposition were AD+NS vs. AD+Dex, 1.0 ± 0.08 vs. 0.85 ± 0.10 AU, *p* = 0.02, and AD+Dex vs. AD+Dex+Y, 0.85 ± 0.10 vs. 1.07 ± 0.08 AU, *p* < 0.001 (Figure 2C). The Abeta deposition areas in the hippocampus DG in different groups were as follows: AD+NS vs. AD+Dex, 1.00 ± 0.32 vs. 0.37 ± 0.14 AU, *p* < 0.001; AD+Dex vs. AD+Dex+Y, 0.37 ± 0.14 vs. 0.90 ± 0.23, *p* = 0.001. The mean gray values of Abeta deposition were AD+Dex vs. AD+Dex+Y, 0.90 ± 0.11 vs. 1.05 ± 0.07 AU, *p* = 0.03, *n* = 6 in each group (Figure 2D). This finding reveals that dexmedetomidine delays hippocampal Abeta deposition in 5xFAD mice through an α2 receptor-dependent mechanism.

### Dexmedetomidine reduces 5xFAD mouse hippocampal inflammatory responses

**Figure 3:**
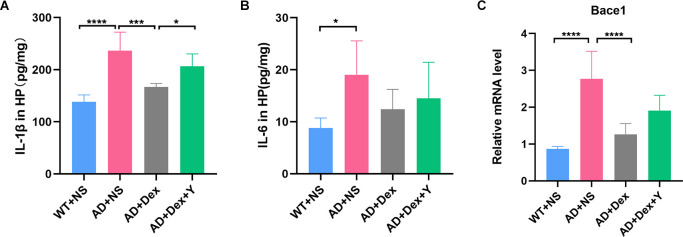
Panels **(A,B)** show ELISA data of IL-1β and IL-6 expression levels in the hippocampus (*n* = 6 in each group). **(C)** The expression level of Bace1 in qRT-PCR (*n* = 6 in each group). *p < 0.05, ***p < 0.001, ****p < 0.0001.

Enzyme-linked immunosorbent assay showed that the levels of IL-6 and IL-1β in 5xFAD mice were significantly higher than those in their littermate WT mice when they were administered NS (IL-6, 8.82 ± 1.89 pg/mg vs. 19.04 ± 6.51 pg/mg, *p* = 0.01; IL-1β, 138.70 ± 12.92 pg/mg vs. 237.00 ± 35.07 pg/mg, *p* < 0.0001; Figures 3A,B). Administration of dexmedetomidine reduced the level of IL-1β in hippocampal tissue (237.00 ± 35.07 pg/mg vs. 167.20 ± 6.50 pg/mg, *p* = 0.0002). The α_2_ receptor antagonist yohimbine counteracted the anti-inflammatory effect of dexmedetomidine (IL-1β: 167.20 ± 6.50 pg/mg vs. 206.30 ± 23.96 pg/mg, *p* = 0.03, *n* = 6 in each group; Figure 3A). IL-6 levels were not different in the three 5xFAD groups (Figure 3B).

**Figure 4:**
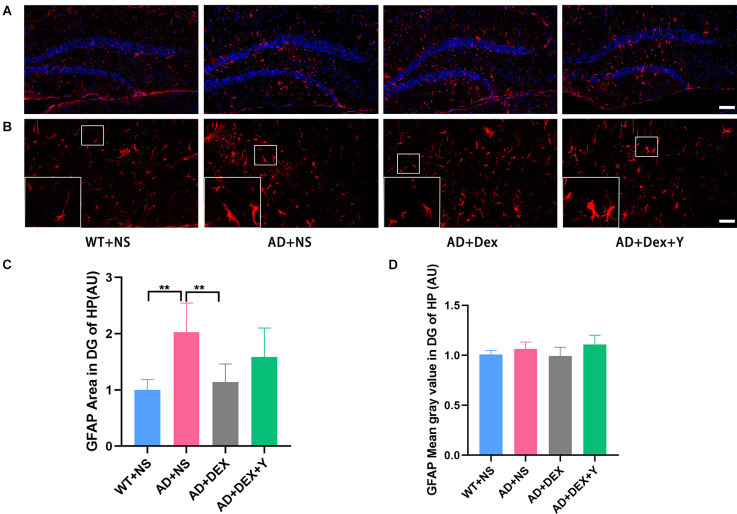
Dexmedetomidine inhibited glial cell activation in the hippocampus DG of 5xFADmice (means ± SD, ANOVA, **p < 0.01). **(A)** Representative images of GFAP-positive staining in the hippocampus DG from WT and 5xFAD mice, scale bars, 100 μm. **(B)** High magnification images of GFAP-positive staining in the hippocampus DG from WT and 5xFAD mice, scale bars, 50 μm. **(C,D)** The bar graph represents GFAP expression levels in the hippocampus DG.

**Figure 5:**
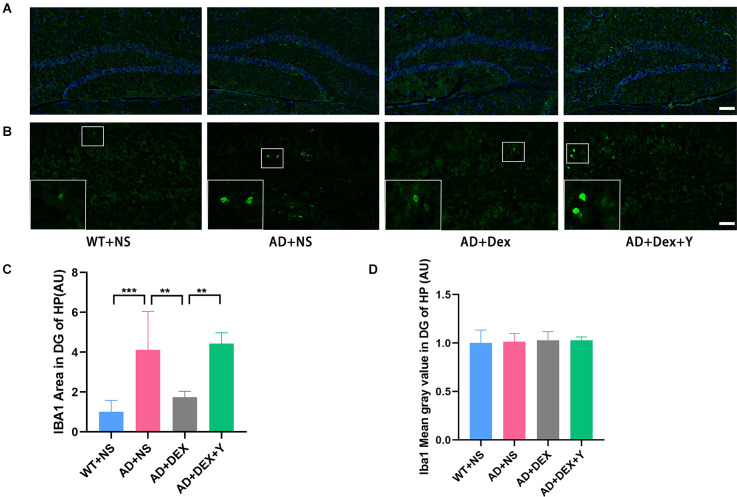
Dexmedetomidine inhibited glial cell activation in the hippocampus DG of 5xFAD mice (means ± SD, ANOVA, **p < 0.01, ***p < 0.001). **(A)** Representative images of IBA1-positive staining in the hippocampus DG from WT and 5xFAD mice, scale bars, 100 μm. **(B)** High magnification images of IBA1-positive staining in the hippocampus DG from WT and 5xFAD mice, scale bars, 50 μm. **(C,D)** The bar graph represents IBA1 expression levels in the hippocampus DG (*n* = 6 in each group).

DG is more heavily regulated by various environmental and neurotrophic factors than other areas of the hippocampus (Yamashima et al., 2004; Lee and Son, 2009). So, we assessed the activation of astrocytes and microglia in hippocampus and area DG by immunostaining respectively using glial fibrillary acidic protein (GFAP) and ionized calcium-binding adapter molecule 1 (Iba-1). In the experiment, we found the level of GFAP was increased in the 5xFAD group of animals than that in littermate wild-type mice, and the administration of dexmedetomidine reduced the level of GFAP in the hippocampus DG (Figures 4A,B). The GFAP areas in the hippocampus DG in different groups were as follows: WT+NS vs. AD+NS, 1.00 ± 0.18 vs. 2.03 ± 0.52, *p* = 0.002; AD+NS vs. AD+Dex, 2.03 ± 0.52 vs. 1.14 ± 0.32 AU, *p* = 0.006. The GFAP areas of hippocampus DG in the AD+Dex+Y group increased compared with that in the AD+Dex group, although not statistically significant (Figures 4C,D). A significant increase of Iba1-positive microglial cells was detected in AD mice than that in littermate wild-type mice, administration of dexmedetomidine reduced the Iba1-positive microglial cells in the hippocampus DG (Figures 5A,B). The Iba1 areas in the hippocampus DG in different groups were as follows: WT+NS vs. AD+NS, 1.00 ± 0.58 vs. 4.12 ± 1.93, *p* < 0.001; AD+NS vs. AD+Dex, 4.12 ± 1.93 vs. 1.73 ± 0.31 AU, *p* = 0.004; AD+Dex vs. AD+Dex+Y, 1.73 ± 0.31 vs. 4.43 ± 0.55 AU, *p* = 0.001, *n* = 6 in each group. The GFAP areas of hippocampus DG in the AD+Dex+Y group increased compared with that in the AD+Dex group, although not statistically significant (Figures 5C,D).

### Dexmedetomidine reverses 5xFAD mouse hippocampal NfκB-P65 phosphorylation and prevents the production of bace1

**Figure 6:**
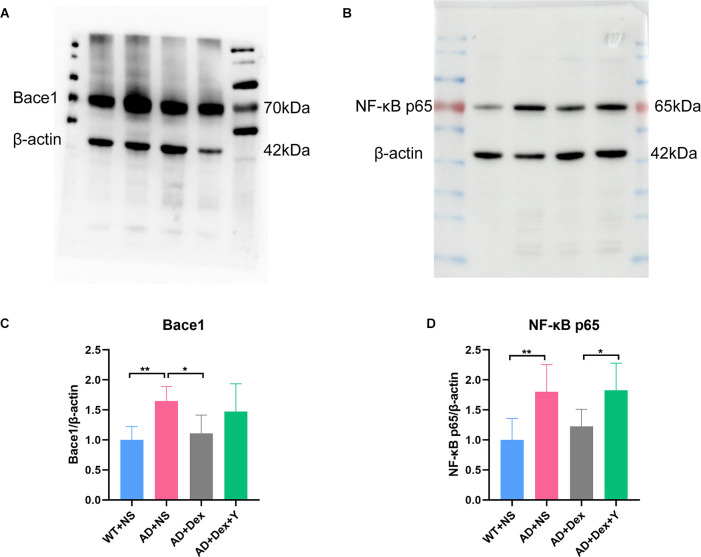
Dexmedetomidine downregulates the expression of Bace1 **(A,C)** and NF-κB p65 (**B,D**; means ± SD, ANOVA, *p < 0.05, **p < 0.01). **(A)** Western blotting data showing Bace1 expression levels in the hippocampus. **(B)** Western blotting data of NF-κB p65 expression levels in the hippocampus. **(C)** The bar graph represents Bace1 expression levels in the hippocampus. **(D)** The bar graph represents NF-κB p65 expression levels in the hippocampus (*n* = 6 in each group).

In the qRT-PCR experiment, we found that the level of bace1 mRNA expression in 5xFAD mice was significantly higher than that in littermate wild-type mice (WT+NS vs. AD+NS, 0.86 ± 0.07 vs. 2.77 ± 0.75, *p* < 0.0001; Figure 3C). Administration of dexmedetomidine reduced the level of bace1 expression in the hippocampus (AD+NS vs. AD+Dex, 2.77 ± 0.75 vs. 1.26 ± 0.30, *p* < 0.0001). Western blot analysis showed that the expression of bace1 in the AD+NS group was significantly increased compared with that in the WT+NS group (WT+NS vs. AD+NS, 1.00 ± 0.22 vs. 1.65 ± 0.24, *p* = 0.003); the expression of bace1 in the AD+Dex group was significantly decreased compared with that in the AD+NS group (AD+Dex vs. AD+NS, 1.11 ± 0.30 vs. 1.65 ± 0.24, *p* = 0.03, *n* = 6 in each group; Figures 6A,C).

There was a significant difference in the expression of NF-κB-p65 between the WT+NS group and the AD+NS group (WT+NS vs. AD+NS, 1.00 ± 0.36 vs. 1.80 ± 0.45, *p* = 0.004 (Figure 4D). The expression of NF-κB-p65 in the AD+Dex+Y group increased significantly compared with that in the AD+Dex group (AD+Dex vs. AD+Dex+Y, 1.27 ± 0.28 vs. 1.83 ± 0.45, *p* = 0.04, *n* = 6 in each group; Figures 6B,D). This finding reveals that dexmedetomidine acts on bace1 through the NF-κB signaling pathway in the hippocampus of 5xFAD mice through an α2 receptor-dependent mechanism.

## Discussion

5xFAD mice are a classic model of AD and express human APP under the control of the murine Thy-1 promoter (Oakley et al., 2006). Extracellular amyloid plaques can be detected as early as 2 months in the hippocampus, and amyloid plaques and cognitive decline worsen with age (Richard et al., 2015). In this study, we found that WT mice performed better than 5xFAD mice in the Y-maze and Morris water maze. Dexmedetomidine treatment improved the spatial and learning memory performance of 5xFAD mice. Biochemical experiments showed that 5xFAD mice treated with dexmedetomidine had less Abeta deposition than those treated with NS. The expression levels of GFAP, IBA1, bace1, NF-κB p65, and IL-1β were downregulated. However, yohimbine neutralized the effects of dexmedetomidine. The present study demonstrated that dexmedetomidine exerts an anti-inflammatory effect *via* the α_2_ adrenoceptor to alleviate cognitive function decline in 5xFAD mice.

AD is a complex neurodegenerative disease with two main pathological features, amyloid plaques, and neurofibrillary tangles. In recent years, an increasing number of studies have started to focus on neuroinflammation in AD (Howard et al., 2020; Zhang F. et al., 2021), and many studies have pointed out the important role of inflammation in the progression of AD (Holmes, 2013; McAlpine et al., 2021; Milner et al., 2021). Inflammation in the brains of AD patients is a typical double-edged sword, and activation of microglia and astrocytes can eliminate debris and protein aggregates; in contrast, over-activated glia produces inflammatory factors leading to neuronal system damage (Schwartz and Deczkowska, 2016). Pascoal’s team proposed that amyloid plaques, neurofibrillary tangles, and neuroinflammation interact with each other and together promote the development of AD (Pascoal et al., 2021). Persistently activated microglia have been found in the AD brain and are associated with overexpression of IL-6, IL-1β, and NF-κB (Bales et al., 1998). It was reported that IL-6 is involved in dementia (Koyama et al., 2013). Griffin et al. (1998) found that IL-1β promotes the synthesis of β-amyloid precursor protein and induces the production of Aβ. Furthermore, Chen’s report indicates that NF-κB p65 overexpression leads to upregulated β-secretase cleavage and Abeta production (Chen et al., 2012). Hence, anti-inflammatory effects have therapeutic potential.

Dexmedetomidine not only has anti-inflammatory effects (Li et al., 2018) but also can improve cognitive impairment caused by various reasons (Deiner et al., 2017; Alam et al., 2018). Animal experiments have shown that dexmedetomidine can reduce the inflammatory response by reducing IL-1β and NF-κB levels *via* α2 adrenoceptors, thereby improving the cognitive decline caused by inflammation (Li et al., 2020). In addition, dexmedetomidine can protect against Aβ_1–42_-induced hippocampal neuronal apoptosis and cognitive impairment in mice (Sun et al., 2020). Furthermore, dexmedetomidine could also improve cognitive function in aged rats, and it may be associated with Abeta (Zhang et al., 2018). Some clinical trials have shown that reducing amyloid plaques fails to improve cognition, leading some studies to conclude that Abeta has little effect on AD (Cummings et al., 2019; Egan et al., 2019). There is growing evidence that Abeta may influence synaptic activity, tau pathology, and neuroinflammation (Pereira et al., 2021).

The effect of dexmedetomidine on cognitive function and Abeta deposition in AD is still unknown, so we investigated the efficacy of dexmedetomidine in 5xFAD mice and explored the possible mechanism. Alzheimer’s disease is a chronic progressive disease, so we need to choose a long-term administration time to affect the course of the disease. European Medicines Agency revealed that no accumulation in patients when dexmedetomidine was infused for 14 days (Keating, 2015), therefore, by administrating dexmedetomidine for 14 days not only the action time of the drug is guaranteed, but also there is no accumulation in mice in subsequent behavioral experiments. Our study shows that dexmedetomidine can prevent Abeta deposition by regulating NF-κB-bace1, reduce neuroinflammation resulting from Abeta, and prevent cognitive decline in 5xFAD mice.

Dexmedetomidine is a highly selective α2 adrenergic receptor agonist, and α2 adrenoceptors are distributed on circulating and tissue immunocytes, so dexmedetomidine can affect the innate immune response through α2 adrenergic receptors (Quatrini et al., 2018). In ischemia-reperfusion injury in the heart, dexmedetomidine activation of α2 adrenoceptors downregulates toll-like receptor 4 (TLR4) expression and then regulates NF-κB (Sun et al., 2020); therefore, α2 adrenoceptors may regulate bace1 through the TLR4-NF-κB signaling pathway, affecting Abeta deposition. Yohimbine has an extremely high affinity for α2 adrenoceptors and can counteract the effect of dexmedetomidine through α2 adrenoceptors (Gao et al., 2019).

Our study has limitations. Our results indicate dexmedetomidine inhibits neuroinflammation in 5xFAD mice, but the mechanism is not investigated. Reports reveal that dexmedetomidine can improve sleep disorders (Zhang Y. et al., 2021), and sleep disorders play an important role in AD (Irwin and Vitiello, 2019), maybe dexmedetomidine can alleviate AD by improving sleep disorders.

In conclusion, dexmedetomidine alleviates cognitive decline, prevents the production of Abeta, and inhibits neuroinflammation in 5xFAD mice. These findings demonstrated that dexmedetomidine may be a potential drug for the treatment of AD. This conclusion may benefit the clinical use of the drug in patients at risk for cognitive impairment.

## Data Availability Statement

The original contributions presented in the study are included in the article, further inquiries can be directed to the corresponding author.

## Ethics Statement

The animal study was reviewed and approved by Ethics Committee for Animal Research of Central South University.

## Author Contributions

EW designed and supervised the study. S-mL, L-yL, L-zG, NC, and Y-fW performed the experiments. S-mL and LW wrote the manuscript draft. All authors contributed to the article and approved the submitted version.

## Funding

This work was financially supported by the National Key Research and Development Program of China (No: 2020YFC2005300) and the Youth Program of National Natural Science Foundation of China (grant number 81601728).
